# Properties of Plastic-Based Composite Panels Manufactured from Municipal Waste Under Accelerated Weathering as Potential Paving Slabs

**DOI:** 10.3390/polym17222998

**Published:** 2025-11-11

**Authors:** Chatree Homkhiew, Pruttipong Pantamanatsopa, Sriwan Khamtree, Chainarong Srivabut, Worapong Boonchouytan, Surasit Rawangwong, Salim Hiziroglu

**Affiliations:** 1 Materials Processing Technology Research Unit, Faculty of Engineering, Rajamangala University of Technology Srivijaya, Muang District, Songkhla 90000, Thailand; chatree.h@rmutsv.ac.th (C.H.); pruttipong.p@rmutsv.ac.th (P.P.); worapong.b@rmutsv.ac.th (W.B.); 2 Department of Industrial Technology, Faculty of Industrial Technology, Songkhla Rajabhat University, Muang District, Songkhla 90000, Thailand; sriwan.kh@skru.ac.th; 3 Department of Industrial Engineering, Faculty of Engineering, Rajamangala University of Technology Srivijaya, Muang District, Songkhla 90000, Thailand; surasit.r@rmutsv.ac.th; 4 Department of Natural Resource Ecology & Management, Oklahoma State University, Stillwater, OK 74078-6013, USA; salim.hiziroglu@okstate.edu

**Keywords:** polymer composite, municipal waste, paving slabs, mechanical properties, foil bags, plastic bags, aluminum foil

## Abstract

This research examined the mechanical, physical, thermal, and durability properties of plastic-based composites made from MSW, namely ultra-high-temperature (UHT) cartons, plastic bags, aluminum foil, and foil bags under both unweathered and accelerated weathering conditions to evaluate their potential as paving slab materials. Composite samples with varying mixing ratios were fabricated and tested based on an experimental design. Statistical analyses using one-way ANOVA confirmed the significant effects of composition on material performance (*p* < 0.05). The results demonstrated that the mixing ratio markedly influenced mechanical properties. The composite containing 50 wt% UHT carton and 50 wt% foil bags (U50F50) achieved the highest modulus of rupture (121.20 MPa) and modulus of elasticity (2.98 GPa), as well as compressive strength (28.56 MPa), compressive modulus (2.12 GPa), screw withdrawal resistance (54.25 MPa), and hardness (66.25). Under accelerated weathering, all of the composites showed moderate reductions in strength (10 to 30%) due to plastic degradation and surface cracking. In contrast, the composites containing high paperboard fractions (U80P15A5) exhibited greater WA (3.55%) and TS (3.04%), attributed to the hydrophilic nature of cellulose. The inclusion of foil bags effectively reduced WA and TS by limiting moisture penetration. Density measurements demonstrated a gradual increase (0.99 to 1.05 g/cm^3^) with higher foil content, while accelerated weathering induced an average 10% density reduction. Abrasion resistance improved in foil-rich composites, with U50F50 showing the lowest weight loss (8.56 to 14.02%), confirming its superior structural integrity under mechanical wear. Thermal analysis indicated low conductivity values (0.136 to 0.189 W/m·K), demonstrating favorable insulation performance compared to conventional paving materials. However, higher foil bag fractions enhanced heat conduction, balancing mechanical strength with thermal functionality. Overall, MSW-derived composites containing 30 to 50 wt% foil bags exhibited optimal mechanical durability, abrasion resistance, and thermal stability, making them promising candidates for sustainable paving slab production with low environmental impact and enhanced service life.

## 1. Introduction

The global reliance on plastics has led to a significant surge in waste generation, posing critical environmental and socioeconomic challenges [1]. Due to their durability, versatility, and cost-effectiveness, plastics are used extensively across multiple industries, including packaging, construction, automotive, and consumer goods [2,3,4]. However, these same attributes contribute to their persistence in the environment, with millions of tons of plastic waste accumulating in landfills and ecosystems each year, causing adverse effects on the environment [5,6]. In response, scientific and industrial communities have increasingly focused on the development of plastic composites engineered using materials formed by combining plastics with fillers or reinforcements that not only improve material performance but also enhance sustainability through the incorporation of recycled content [7,8]. The exponential increase in municipal solid waste (MSW) generated by urban populations has also emerged as a pressing environmental and economic concern globally [2,9,10]. Among the diverse waste streams, post-consumer packaging materials such as ultra-high temperature (UHT) milk cartons, plastic bags, aluminum foil, and multilayer foil bags constitute a significant portion due to their widespread use and composite nature [11,12,13]. These materials are particularly challenging to recycle because of their heterogeneous structure, comprising plastics, paperboard, and metals laminated together [7,14,15]. It is a well-known fact that conventional disposal methods, including landfilling and incineration, pose long-term environmental hazards such as greenhouse gas emissions, leachate contamination, and resource depletion [16]. In response to global sustainability goals and circular economy principles, there is an urgent need to develop value-added applications for such complex waste materials, particularly by converting them into durable, reusable products [17,18].

Despite growing awareness and regulatory efforts to reduce plastic pollution, the recycling of multilayer packaging materials remains underdeveloped, primarily due to technological and economic constraints associated with their separation and classification process [19]. Current recycling technologies often overlook the potential of mixed MSW streams as viable raw materials for composite manufacturing [9,20]. However, recent advances in thermomechanical processing, especially compression molding, have shown promise in transforming heterogeneous plastic-based waste into structural materials without the need for extensive preprocessing [4,21]. This paradigm shift presents an opportunity to explore MSW as a raw resource for manufacturing green composite materials, aligning with both waste valorization strategies and sustainable construction practices [22,23]. Nevertheless, several critical knowledge gaps hinder the practical implementation of such technologies. First, comprehensive studies examining the mechanical, physical, as well as thermal properties of composite materials derived directly from unseparated post-consumer waste mixtures remain scarce [14,24,25]. Recent research projects either focus on single-type polymers or require costly separation techniques. Second, optimization of material composition using statistical tools like Analysis of Variance (ANOVA) to assess the influence of various waste types and proportions on composite performance remains limited [2,3,4,9]. Furthermore, the application of these recycled composites in real-world construction contexts, such as sidewalk paving slabs, has not been thoroughly investigated, despite their potential to meet basic structural and environmental standards [3,26,27].

This study addresses the existing knowledge gaps by investigating the feasibility of utilizing selected municipal solid waste components for manufacturing plastic-based composites [28,29]. The most promising composition was then developed into prototype sidewalk paving slabs to demonstrate its practical applicability and structural viability within the perspective of theoretical and practical contributions [15,30]. Theoretically, it advances understanding of material behavior in mixed-waste composite systems, particularly the synergistic effects of polymer-metal paper blends under thermal compression [31,32]. Practically, it provides a scalable approach to upcycle difficult-to-recycle municipal solid waste into durable construction materials, contributing to sustainable urban development and circular economy models [8,33]. The originality of this work lies in its holistic integration of real-world waste streams and functional prototyping, positioning it as a model for sustainable materials engineering using urban waste [34,35].

Accordingly, the main objective of this work was to investigate the effects of varying plastic waste mixing ratios on the mechanical, physical, and thermal properties of plastic-based composites. Municipal solid waste, including ultra-high-temperature processing (UHT), plastic bags, aluminum foil, and foil bags, was processed using compression molding to form composite samples. However, the novelty and distinction of this research lie in utilizing mixed municipal plastic waste for construction applications while also providing a scalable and environmentally sustainable pathway for waste valorization.

## 2. Materials and Methods

### 2.1. Materials

Ultra-high-temperature processing (UHT) cartons, plastic bags, aluminum foil, and foil bags were collected from the Mueang District municipal solid waste landfill site (Songkhla, Thailand). The UHT carton is a food processing technology that sterilizes liquid food by heating it above 140 °C, the temperature required to kill bacterial endospores, for 2 to 5 s. Plastic bags and foil bags are defined as thin-film bags typically made from materials such as polyethylene (PE), commonly used for various purposes, including agricultural products, shopping, and medical waste. Also, aluminum foil is defined as a thin-rolled sheet of alloyed aluminum, with thicknesses ranging from about 4 to 150 μm, primarily used for wrapping and packaging food products.

### 2.2. Preparation of the Composite Samples

**Figure 1:**
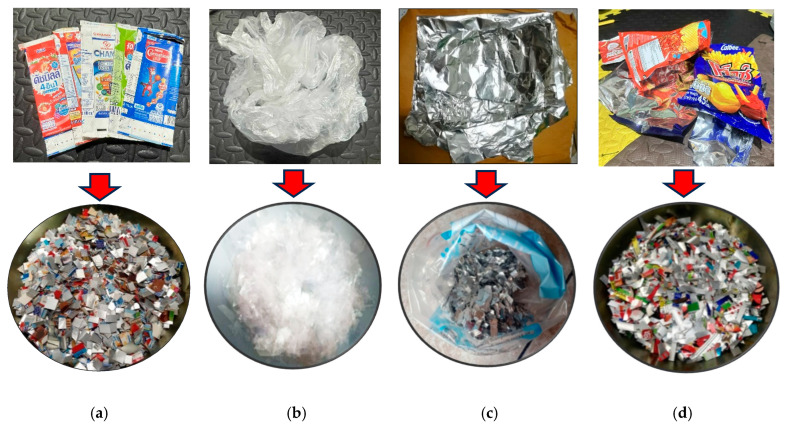
Municipal solid waste (MSW) using in this research: (a) ultra-high-temperature processing (UHT) carton, (b) plastic bags, (c) aluminum foil, and (d) foil bags.

The processing conditions of composite blends are displayed in Table 1. All of the composites were prepared in two stages, namely preparing the plastic composites components in the first stage, followed by washing of the UHT cartoons, plastic bags, and aluminum foil, with water before drying under the sun for 8 h to remove moisture content. Furthermore, these components were made into smaller pellets using machine cutting based on shearing to break the raw materials into smaller pieces 5 mm in size, after which they were dried in an oven at 50 °C for 24 h before composite mixing, as shown in Figure 1. The second stage is preparing the hot compression molding process. The composition levels of UHT: 50, 60, 70, 80, and 100 wt%, plastic bags: 5 and 15 wt%, aluminum foil: 5 and 15 wt%, and foil bags: 30, 40, and 50 wt%, were determined following the experimental design. All of the ratios were placed using a hot compression molding machine with a compression method from the top direction, having a temperature of 190 °C at a pressure of 2500 psi for 15 min with a sequence of pre-heating, compressing, and cooling, respectively. Finally, the composite specimens were machined, complying with American Society for Testing and Materials (ASTM) standards, prior to any tests being carried out [2,7].

### 2.3. Characterizations of Composite Samples

#### 2.3.1. Flexural Test of the Samples

The modulus of elasticity (MOE) and modulus of rupture (MOR) of the samples were measured to evaluate their flexural properties. The three-point bending test of the samples was carried out according to the ASTM D790 standard [36] employing a Mechanical Universal Testing Machine, Model NRI-TS500-50 from Narin Instrument Co., Ltd., Samut Prakan, Thailand. The machine parameters were set to a crosshead speed of 2 mm/min and a span of 80 mm. Specimens with nominal dimensions of 13 mm (width) × 100 mm (length) × 4.8 mm (thickness), with five replications for each formulation, were kept at a room temperature of 25 °C. The MOR and MOE values of the samples were calculated using Equations (1) and (2) below:

(1) $$\mathrm{MOR}\;\left(\mathrm{MPa}\right)\;=\;\frac{3P_{\max}}{2bd^{2}}$$

where *P*_max_ is the ultimate load (N), *L* is the span distance of support (mm), *b* is the breadth of cross-section of the samples (mm) and *d* is the depth of the samples (mm).

(2) $$\mathrm{MOE}\;\left(\mathrm{GPa}\right)=\frac{P_{\mathrm{pl}}L^{3}}{4\delta_{\mathrm{pl}}bd^{3}}$$

where *P_pl_* is the incremental load (N), *L* is the span distance of support (mm), and *δ_pl_* is the incremental bending distance (mm) in the range where the relation is linear, *b* is the breadth of cross-section of the samples (mm) and *d* is the depth of the samples (mm).

#### 2.3.2. Compressive Test of the Samples

The compressive strength (CS) and modulus (CM) of plastic-based composites were investigated. Specimens with dimension of 4.8 mm (width) × 9.6 mm (length) × 4.8 mm (thickness) were prepared for these tests. The samples were tested according to ASTM D 6108-97 [37] using the Mechanical Universal Testing Machine, Model NRI-TS500-50 from Narin Instrument Co., Ltd., Samut Prakan, Thailand, with a constant displacement rate of 0.5 mm/min. Five replications of each formulation were tested at a room temperature of 25 °C and their CS and CM average values were reported.

#### 2.3.3. Direct Screw Withdrawal Test of the Samples

The direct screw withdrawal test (DSW) of samples was carried out in accordance with ASTM D1037 standard [38] using a Mechanical Universal Testing Machine, Model NRI-TS500-50 from Narin Instrument Co., Ltd., Samut Prakan, Thailand. Wood screws with a diameter of 4.18 mm and a threaded length of 50 mm were used to hold the threaded screws with composite samples before sample testing. The crosshead speed for screw withdrawal was set at 2 mm/min. The nominal dimensions of specimens were 50 mm (width) × 50 mm (length) × 4.8 mm (thickness). All of the samples were tested at room temperature (25 °C) with five replications. The screw withdrawal strength was then calculated using Equation (3) as follows:

(3) $$\mathrm{DSW}\;(\mathrm{MPa})\;=\frac{\mathrm{Pmax}}{{\mathrm{dl}}_{p}}$$

where *P_max_* is maximum load (N) required to withdraw a screw from the specimen, *d* is diameter (mm) of a wood screw, and *l_p_* is depth (mm) of the screw penetration in specimen.

#### 2.3.4. Hardness Test of the Samples

**Figure 2:**
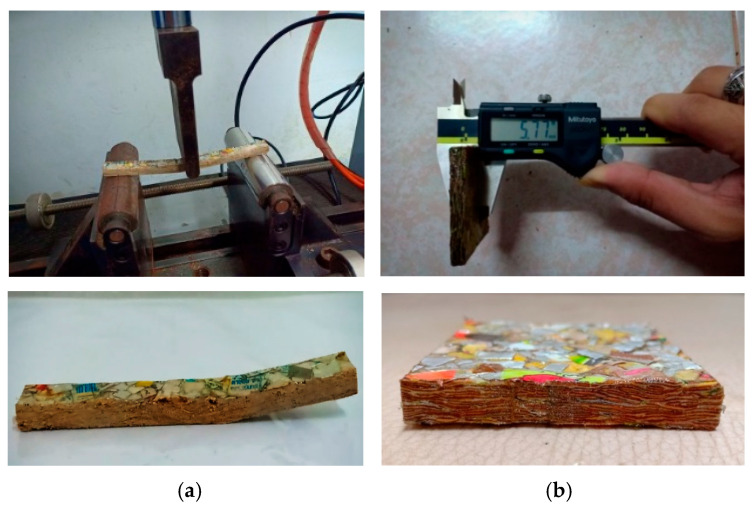
Sample preparation and testing process of some mechanical and physical properties: (a) flexural test and (b) WA and TS tests.

The surface strength of composites was evaluated based on the hardness measurement. The composite samples were carried out in accordance with ASTM D2240 [39] using a mechanical Shore D Durometers, Model GS-702G from Teclock Corporation, Nagano, Japan. Each sample was tested at five different points on the surface. Five replications of the specimens with dimensions of 30 mm (width) × 30 mm (length) × 4.8 mm (thickness) were tested at a room temperature of 25 °C. Sample preparation and the testing process are shown in Figure 2a.

#### 2.3.5. Dimensional Stability Test of the Samples

The water absorption (WA) and thickness swelling (TS) of samples were conducted accordance to the ASTM D570 standard [40] on rectangular specimens with nominal dimensions of 10 mm (width) × 20 mm (length) × 4.8 mm (thickness). Initially, the weight and thickness of each sample were measured before they were soaked in water. Subsequently, the samples of each formulation were soaked in distilled water for 90 days at a room temperature of 25 °C until the samples were saturated. After that, all of the samples were removed, and the excessive water was rinsed before they were remeasured. The average values of the results were determined from five replications. The composite sample preparation and testing process is displayed in Figure 2b. The percentages of WA and TS values of the samples were calculated according to Equations (4) and (5) as follows:

(4) $${\mathrm{WA}}_{t}(\%)\;=\;\frac{W_{t}\;-\;{\;W}_{0}}{W_{0}}\;\times \;100$$

where *WA*_t_ is the water absorption in percentage, and *W_t_* and *W*_0_ are the specimen weights before and after immersion (g).

(5) $${\mathrm{TS}}_{t}(\%)=\frac{T_{t}\;-\;T_{0}}{T_{0}}\;\times \;100$$

where *TS_t_* is the thickness swelling in percentage, and *T_t_* and *T*_0_ are the specimen thickness before and after immersion (mm).

#### 2.3.6. Density Values of the Samples

The standard density of the samples was compared with that of distilled water (approximately 1000 kg/m^3^), which can serve as a reference for evaluating product performance. This standardized method is found using Archimedes’ principle in accordance with ASTM D792 [41] using an Electronic densimeter, Model MD-300S from Alfa Mirage Co., Ltd., Osaka, Japan. The apparent loss of weight of a fully immersed sample is measured to determine the displaced water volume. The specific temperature for the density test of composites in distilled water ranged from 23 to 25 °C. Five replications of the samples with dimensions of 10 mm (width) × 10 mm (length) × 4.8 mm (thickness) were determined. The actual density (ρa) values of the samples were then calculated using Equation (6).

ρa = ρw × Wa/(Wa − Ww)(6)

where *ρw* is the density of distilled water and *Wa* and *Ww* are the specimen’s weight in air and water, respectively.

#### 2.3.7. Abrasion Test of the Samples

The abrasion of composites materials was evaluated based on mechanical contact between samples and environmental. The composite samples were tested based on ASTM D1044 standard [42] using an abrasion tester, Model QC-619H from Gotech Testing Machines Co., Ltd., Taichung, Taiwan, under the conditions of 500 g load and speed 50 r/min for 20, 40, and 60 s. Initially, the samples were cut into a circle with a diameter of 110 mm with a hole of 7 mm in the center. The weight of each specimen was measured before they were fixed to the table and the abrasion wheel was set as the moving arm on the abrasion tester. During the abrasion test, the wheel slid with cycle motion under the above conditions. After that, all of the specimens were removed from the tester and they were reweighted. The wear volume was calculated by measuring the weight before and after of the abraded composite surfaces on each condition under room temperature (25 °C).

### 2.4. Thermal Conductivity of the Samples

The thermal conductivity of the samples was measured to evaluate their overall resistance as a function of different mixing contents. The specimens were tested in accordance with ISO 8301:1991 standard [43] using the HFM 436/3/1E Lambda, Model NETZSCH Group, Selb, Germany. Five replications of specimens with nominal dimensions of 10 × 10 × 4.8 mm^3^ cut from the panels were exposed to temperature levels ranging from −20 to 60 °C. Random fluctuations in the thermal conductivity of natural materials typically result in standard errors of approximately 5%. The reported values represent the averages of thermal conductivity and thermal resistance analyses.

### 2.5. Accelerated Weathering of the Samples

Accelerated weathering of the samples was carried out using a QUV- accelerated weathering tester, Model QUV spray, Q-lab Corporation, USA following the ASTM G 154 standard [44] cycle type I. All of the conditions were set for the fluorescent bulb UVA with 0.89 W/m^2^ and a wavelength of 340 nm, with cycles of UV irradiation for 8 h followed by 1 min spray of de-ionized water, then 1 h of condensation. The composite samples were subjected to the aging process for durations of 7 days at a temperature of 50 °C.

### 2.6. Visual Surface Analysis of the Samples

The surfaces of composite layers in the samples were observed regarding the fractured surface, interlayer structure, distribution phase, and changes on the surfaces using optical microscopy, Model Zeiss Axioskop, Oberkochen, Germany. Before the visual observation of their surfaces, all of the samples were dried in a convection oven at a temperature of 50 °C for 24 h. The composite micrographs were taken from their surface at magnification levels of 100 and 150×.

### 2.7. Statistical Analysis

Municipal solid waste based on different mixing ratios, was analyzed using a statistical method. One-way analysis of variance (one-way ANOVA) was used to determine whether there were any statistically significant differences between the means of four or more independents groups. The statistical analyses on the mechanical and physical properties of plastic composites were evaluated using ANOVA with 5% significance level (*α* = 0.05).

## 3. Results and Discussion

### 3.1. Flexural Properties of the Samples as a Function of Weathering and Mixing Ratios

The modulus of rupture (MOR) and modulus of elasticity (MOE) values of the samples containing different amounts of UHT carton, plastic bags, aluminum foil, and foil bags are displayed in Table 2. Both the MOR and MOE are critical parameters for evaluating the performance of paving slab products for practical applications. In general, the enhancement of MOR and MOE is influenced by the mixing ratio, while the type of reinforcing agent employed also exerts a significant effect on these properties [4,7]. The main effect of municipal solid waste contents was highly significant on the flexural properties at the 5% significance level (*p* < 0.05). The results are verified by the statistical analysis of one-way ANOVA. In addition, the deterioration of paving slab products under environmental exposure significantly influenced their flexural properties [3,4,9].

**Figure 3:**
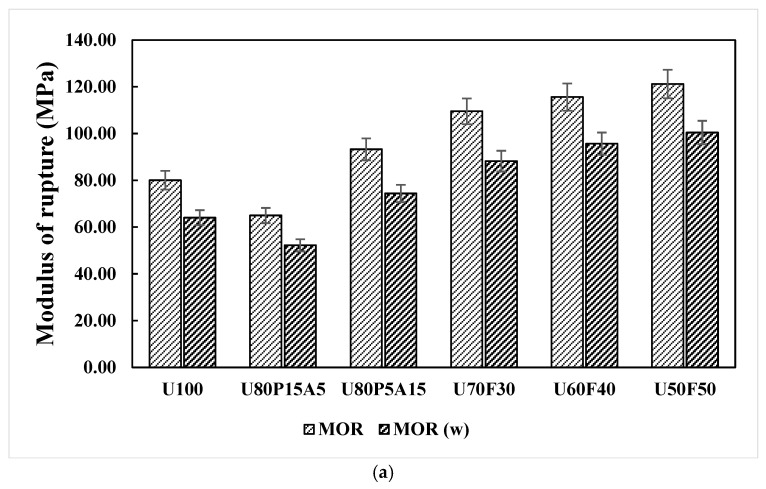

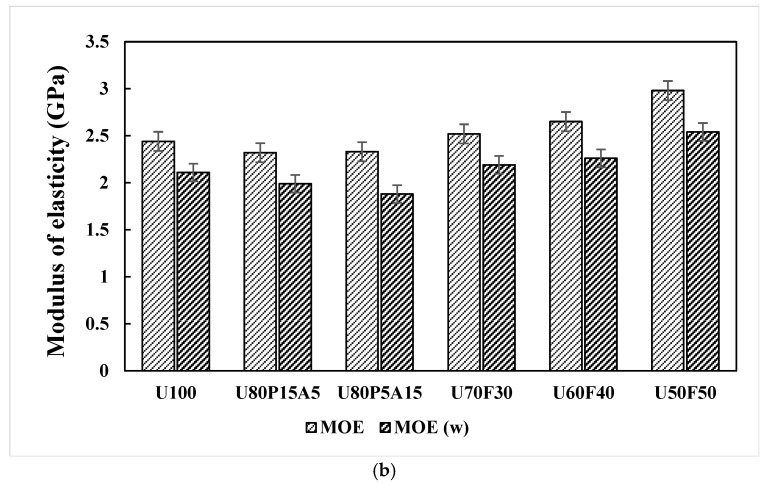
Flexural responses of plastic composites with municipal solid waste contents: (a) modulus of rupture (MOR) and (b) modulus of elasticity (MOE).

As seen in Figure 3a,b, the MOR and MOE values of the samples in unweathered and weathered conditions were investigated, and it was observed that higher MOR and MOE values were obtained in the unweathered samples, ranging from 46.96 to 121.20 MPa and 2.32 to 2.98 GPa. In contrast, the composites subjected to accelerated weathering exhibited reduced MOR and MOE values that ranged from 52.22 to 100.44 MPa and 1.88 to 2.54 GPa, respectively. The flexural properties of polymer composites improved by adding 30 to 50 wt% foil bags to their content. It is evident that the unweathered samples made with U50F50 had enhanced MOR and MOE values compared to those of the composite samples, with values of 121.20 MPa and 2.98 GPa. The positive influence of reinforcement content on the properties of the samples can be attributed to the strong interfacial interactions between the matrix and the reinforcing materials, primarily through hydrogen bond formation and the development of wrapping or adhesive structures within the composites [2,9]. Similarly, under accelerated weathering conditions, the lower MOR and MOE values of 100.44 MPa and 2.54 GPa were recorded for the samples containing UHT carton at 50 wt% and foil bags at 50 wt%, representing 18.2 and 15.1% reduction compared with the unweathered composite samples. This behavior can be attributed to the deterioration of the composites under accelerated weathering. During exposure, leaching of water-soluble constituents from the samples [13,28], together with water absorption during spray and condensation cycles, is likely to occur. Furthermore, the MOR and MOE may have been adversely affected by matrix embrittlement caused by chain scission [32,45], as well as by the formation of surface cracks, both of which intensify with aging.

### 3.2. Effects of Mixing Ratios and Weathering on Compressive Strength of the Samples

**Figure 4:**
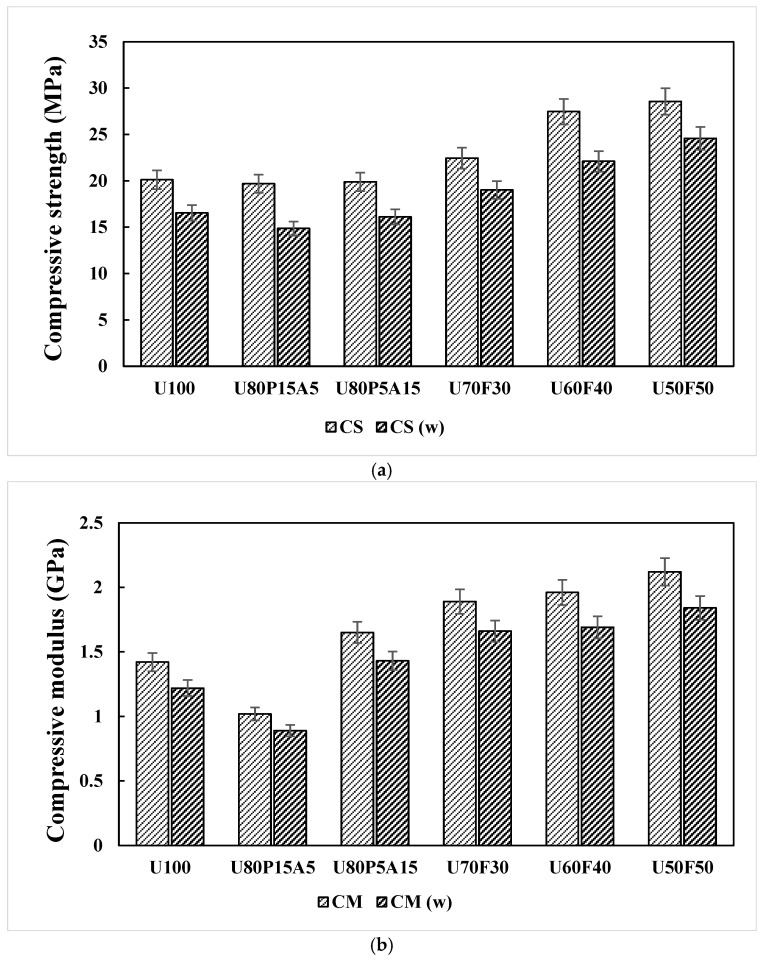
Compressive responses of plastic composites with municipal solid waste contents: (a) compressive strength (CS) and (b) compressive modulus (CM).

Multi-directional compressive values are applied directly to evaluate the paving slab product. The compressive strength (CS) and compressive modulus (CM), including the statistical analysis of one-way ANOVA, are displayed in Table 2. According to the one-way ANOVA, the main effects of composites with different UHT carton, plastic bags, aluminum foil, and foil bags were significant on CS and CM values at the 5% significance level. The increasing trend under the compressive properties of composite materials is illustrated in Figure 4a,b. It was observed that the effect of adding plastic bags at 15 wt% and aluminum foil at 5 wt% in composites produced a sustained decrease in the compressive properties of the samples with a slight change in the amount of reinforcement at equal processing conditions. The CS and CM of all compositions displayed an approximate 2.3 and 8.1% decrease in all of the compositions compared with the control sample (U100). On the other hand, the CS and CM showed an increase of 19.69 to 28.56 MPa and 1.02 to 2.12 GPa, due to the addition of 30 to 50 wt% foil bags in the polymer matrix. The trends of CS and CM recorded in the present work correspond with those reported by Khamtree et al. [10]. Their study showed that the incorporation of greater proportions of reinforcement contents into a plastic matrix leads to elevated CS and CM values in plastic composites. In the case of ratios with high foil bags, all the compositions also showed improved compressive values, probably due to the better dispersion of reinforcement, where the maximum CS and CM values of 28.56 MPa and 2.12 GPa were attained with 50 wt% foil bags (U50F50).

Accelerated weathering was used to evaluate the performance of the samples as a potential paving slab product. The results of compressive strength testing of the samples containing various contents of municipal solid wastes under weathering exposure are also illustrated in Figure 4a,b. Generally, the plastic matrix, UHT cartoon reinforced with plastic bags, aluminum foil, and foil bags exhibited poorer interfacial adhesion compared with that of the composite samples in unweathered conditions, indicating a reduction in the thickness of the interface between the particles and the polymeric matrix [10,16]. The composites were evaluated in comparison with unweathered samples to predict their potential service performance. The decrease in CS and CM of the tested composites ranged from 14.87 to 24.58 MPa and 0.89 to 1.84 GPa, which corresponded to percentage changes of 9.1 and 8.8% compared to those of unweathered composites. This produced a negative effect on the compressive strength of the samples, as explained above [10,22].

### 3.3. Effects of Mixing Ratios and Weathering on the Direct Screw Withdrawal Strength of the Samples

**Figure 5:**
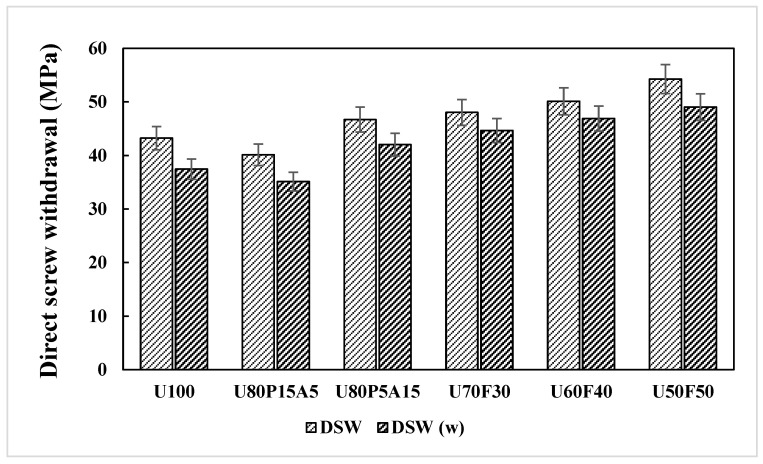
Direct screw withdrawal (DSW) responses of plastic composites with municipal solid waste contents.

The direct screw withdrawal (DSW) strength of the paving slab product was measured by a Universal Testing Machine. Average DSW values under five replications are reported in Table 2. One-way ANOVA was employed to systematically analyze and interpret the data, enabling the identification of patterns, trends, and relationships that provide meaningful insights and support informed decision-making [3,17]. Figure 5 illustrates the relationship between screw withdrawal strength and mixing contents for samples under both unweathered and weathered conditions. It can be seen that the DSW values were found to increase from 42.12 to 54.25 MPa for unweathered and 35.12 to 49.04 MPa for weathered samples, respectively. The maximum DSW value for the unweathered composite samples was observed in the sample having U50F50 (54.25 MPa). Similarly, the weathered composite samples containing 50 wt% UHT carton and 50 wt% foil bags exhibited the highest DSW value (49.04 MPa), which corresponded to percentage changes of 9.6% compared to those of unweathered composites. In contrast, the DSW property under both unweathered and weathered conditions decreases significantly in the composites containing U100 (43.23 and 37.45 MPa) and U80P15A5 (40.12 and 35.12 MPa), because of the chain scission reaction induced by the compressing, while the changes are less pronounced in the foil bags-filled samples. Likewise, the decrease in deformability upon increasing the QUV exposure time is dramatic in the composite samples without foil bags, while it is much less significant in the foil bags-filled samples in the range from 30 to 50 wt%. This can be explained by the homogeneous blend of UHT cartons serving as the matrix and foil bags as reinforcements, both composed of the same plastic substrate, which facilitates strong adhesion between the particles and the matrix [11,20]. Also, this result agrees with Srivabut et al. [9], who concluded that the strong interfacial adhesion between the plastic matrix and reinforcing materials contributes to reduced surface degradation under weathering exposure. Furthermore, the improved homogeneity of the composite structure limits radiation penetration, thereby minimizing molecular chain disruption.

### 3.4. Effects of Mixing Ratios and Weathering on Hardness of the Samples

**Figure 6:**
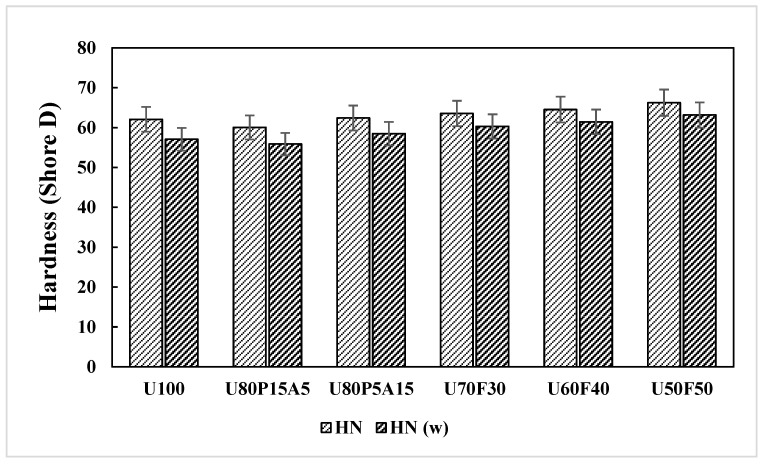
Hardness (HN) responses of plastic composites with municipal solid waste contents.

An in-depth investigation of the surface strength of paving slabs is essential to ensure their structural performance and durability. The hardness (HN) properties for all of the composite samples under both unweathered and weathered conditions are reported in Table 2. Also, the statistical analysis, one-way ANOVA, was used to classify all of the different data [2,4,9]. Overall, the HN values of the composite material increased with increasing reinforcement content, which can be attributed to the enhanced interfacial adhesion between the matrix and reinforcing agent [4,21]. The higher foil bags displayed the increase in HN responses in both unweathered and weathered conditions. The addition of foil bags in the range from 30 to 50 wt% showed a significant effect (*p* < 0.05) on the HN properties. As seen in Figure 6, the highest HN values of 66.25 and 63.17 were determined in the composite sample having U50F50. This can be explained by the high flow value of the plastic matrix, which helps blend both foil bags and UHT carton during the compounding process at a given temperature, thereby giving higher HN properties [2,3,11]. It is evident that the results of this experiment are consistent with previous studies and exhibit a trend similar to that observed in all of the mechanical tests.

It can also be observed that the HN changes are limited during the first 40 h of accelerated weathering, which starts to decrease significantly for longer exposure times (168 h). The HN responses of the weathered samples ranged from 55.90 to 63.17, showing minimal variation compared with the unweathered composites and the control sample (U100), with an overall decrease of approximately 10.2%. Moreover, an explanation can easily be found considering that the foil bags-filled samples have a lower amount of plastic subjected to photooxidative degradation, in which the decrease in HN values upon increasing the irradiation time is smaller in the 50% foil bags-filled samples.

### 3.5. Effects of Mixing Ratios and Weathering on WA and TS of the Samples

**Figure 7:**
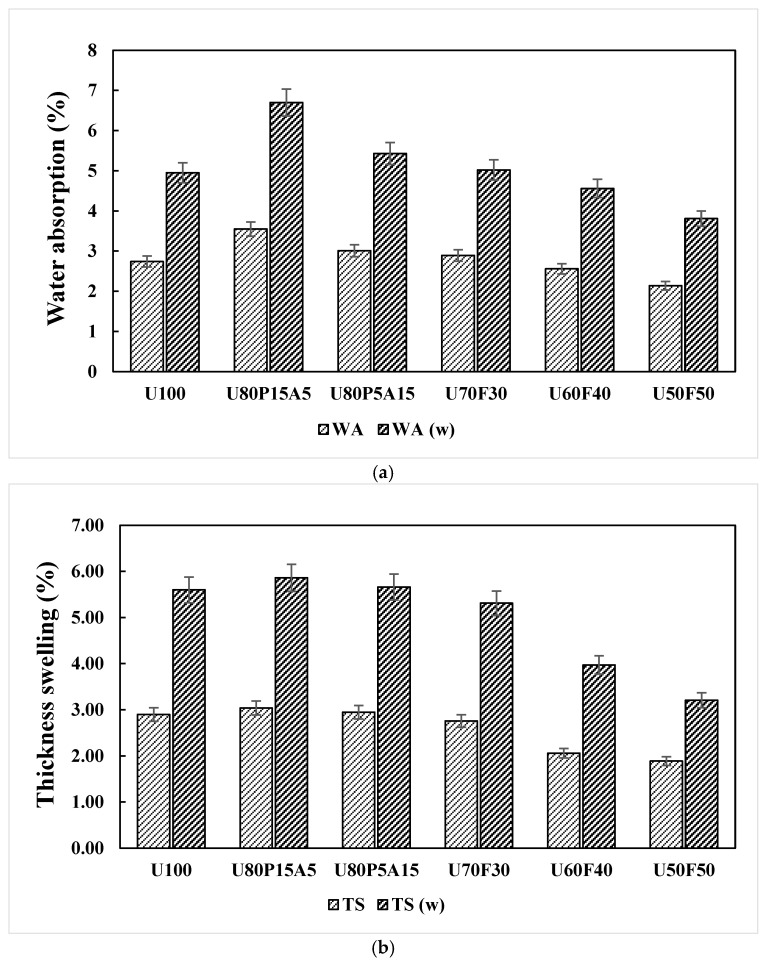
Dimensional stability responses of plastic composites with municipal solid waste contents: (a) water absorption (WA) and (b) thickness swelling (TS) properties.

The long-term water absorption (WA) and thickness swelling (TS) of the composite samples were monitored by full water immersion over a period of 90 days (2160 h), as shown in Table 3. In general, the WA and TS properties increased with the addition of natural fillers such as wood, fiber, and other organic additives, as well as with increasing immersion time until equilibrium conditions were reached. During this period, the plastic matrix exhibited negligible water absorption, whereas the natural fillers contributed significantly to the overall water uptake [26,46]. In this study, the WA and TS values of all composite samples were evaluated under both unweathered and weathered conditions. Average WA and TS values from sample specimens are displayed in Figure 7a,b. It can be seen that the highest WA and TS values were observed in the composite containing 80 wt% UHT carton, 15 wt% plastic bags, and 5 wt% aluminum foil (U80P15A5), with WA and TS values of 3.55% and 3.04%, respectively. This may be attributed to the high proportion of paperboard, the primary component of UHT carton as wood paper layers, which is inherently hydrophilic and thus leads to greater water absorption. Additionally, an increase in the paper content within the composite enhances the number of free hydroxyl (–OH) groups in the cellulose structure, thereby promoting higher water absorption [15,23]. The trends of WA and TS properties recorded in the present research correspond with those reported by Sahu and Gupta [26]. Their study showed that the incorporation of greater proportions of wood components into a plastic matrix leads to elevated water absorption and thickness swelling in plastic-based composites. However, the same plastic contents, with the addition of 30 to 50 wt% foil bags to the composites, significantly (*p* < 0.05) reduced the WA and TS values. Minimizing WA and TS values of 2.145 and 1.895 were determined in a sample having 50 wt% UHT carton and 50 wt% foil bags (U50F50). This is due to the ratio of the UHT carton being replaced by the ratio of foil bags, resulting in less area for water absorption.

Very little change in WA and TS properties was found for all of the formulations, but the percentage of both WA and TS increased for plastic composites with increasing duration of accelerated weathering. Such trends are also shown in Figure 7a,b. It was observed that the U80P15A5 composite exhibited the greatest variations in WA and TS, with increases of approximately 29.6% and 4.8%, respectively. Moreover, the surface of the composite materials subjected to accelerated weathering significantly influenced the increase in WA and TS. The observed increases in these values could be attributed to the degradation of the plastic matrix and the deterioration of interfacial bonding between layers, particularly evident in composites with multiple components, as shown in Table 4. These results could explain the limitations of pavement slab applications and their lifespan extension under optimal conditions [2,4,20].

### 3.6. Effects of Mixing Ratios and Weathering on the Density of the Samples

Density is one of the most important factors in determining the properties of plastic composites [21,47] and is defined as the mass of the material per unit volume, measured according to a standard. Generally, the density of a composite is defined as the density of a material formed by combining the volume fractions of the reinforcement and the matrix, calculated according to the rule of mixtures, which weights the densities of the individual components by their respective volume fractions [8,12]. In filler-reinforced plastic composites, the density depends primarily on the relative proportions of the matrix and reinforcement. The density of the composite constituents, matrix and reinforcement was determined by measuring the specimen’s weight in air and subsequently while suspended in water using a fine wire, with the difference in weight used to calculate the density [20,21].

**Figure 8:**
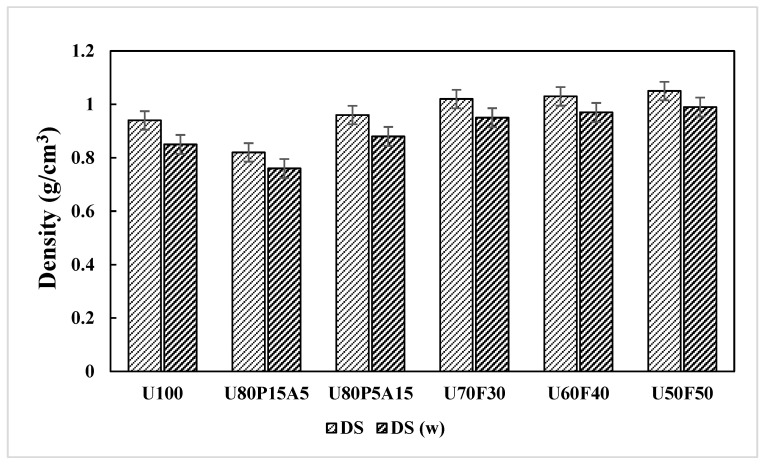
Density (DS) response of plastic composites with municipal solid waste contents.

As seen in Table 5 and Table 6, the surfaces of the samples exposed to accelerated weathering exhibited noticeable changes, including color fading and the formation of a white chalky deposit. The variation in weight during weathering, which significantly (*p* < 0.05) influenced the density values of the composites, is shown in Table 3. It was observed that the density of all composite samples decreased significantly after accelerated weathering, exhibiting an approximate 10% reduction compared with the unweathered specimens. This reduction can be attributed to the leaching of water-soluble components from the samples during weathering, as well as material loss caused by the water spray and condensation cycles. The leaching phenomenon was also evidenced by the previously mentioned color fading. Overall, the observed decrease in weight indicates that leaching was more dominant than water absorption. The present work corresponds with those reported by Sun et al. [13] and Bachchan et al. [16]. Additionally, the decreasing trend in sample density under both conditions is illustrated in Figure 8. The lowest density values were observed in the composites containing U100 and U80P15A5, followed by a gradual increase with the rising foil bag content (30 to 50 wt%). Moreover, the highest density values of 1.05 and 0.99 g/cm^3^ were found in composite materials containing 50 wt% UHT carton and 50 wt% foil bags (U50F50). This can be attributed to the high content and intrinsic density of the foil bag component, which contribute to the overall increase in the density of the plastic composite material.

### 3.7. Effects of Mixing Ratios and Weathering on Abrasion of the Samples

Abrasion testing in various conditions is an important factor in paving slabs applications manufactured from plastic composite materials. Theoretically, the abrasion of plastic-based composites is caused by mechanical contact or exposure to flowing water or particulates. Abrasion results mainly in the localized loss of material from the surface and loosening between the aggregate and paste [17,47,48]. The abrasion resistance of plastic-based composites is influenced by a number of factors, including strong adhesion, surface strength, and properties of supplementary strengthening materials, such as reinforcement materials [22,49]. In this research, the initial and time-dependent weight data for all composite formulations at 20, 40, and 60 s of exposure, along with corresponding percentage weight losses. The statistical significance of differences among samples was confirmed by one-way ANOVA (*p* < 0.05).

Table 7 shows the abrasion properties of plastic composites with different municipal solid waste content under an un-accelerated weathering test. Composite surfaces of the samples before the abrasion test are also displayed in Table 5. A distinct decreasing trend in sample weight with exposure duration was observed for all formulations, indicating progressive degradation and material erosion induced by abrasion. The initial weights of the specimens were relatively consistent, ranging from 21.8 to 22.6 g, confirming uniformity in sample preparation. However, the residual weights decreased considerably after 60 s of testing, with U100 exhibiting the least reduction and U80P15A5 exhibiting the highest. In addition, the relationship between abrasion time and weight loss percentage across different composite formulations was studied. The overall weight loss ranged from 8.56 to 12.96%, signifying variations in degradation resistance based on composition. The highest weight loss was recorded for U80P15A5 (12.96%), while the lowest was found in U50F50 (8.56%). This indicates that increasing the foil bag content enhanced the structural stability of the composites. The presence of foil bag layers within the matrix likely acted as a physical barrier against heat and moisture penetration, slowing down plastic oxidation and chain scission [17,50].

The abrasion resistance of plastic composites with different municipal solid waste content under accelerated weathering is illustrated in Table 8. The surface characteristics of the plastic composites and the specimens are shown in Table 6. Overall, all of the composite samples exhibited measurable weight reduction as abrasion time increased, reflecting gradual material removal due to surface wear and microstructural degradation. The data reveal that weight loss after 60 s of abrasion ranged between 14.02% (U50F50) and 19.78% (U80P15A5). These results demonstrate a clear dependence of abrasion resistance on the relative proportion of UHT carton, plastic bags, and aluminum foil waste constituents. The U80P15A5 composite, containing a relatively high proportion of plastic and aluminum foil bag waste, exhibited the highest weight loss (19.78%). This suggests that the matrix-phase weakening and interfacial debonding between the polymer and reinforcement components accelerated material detachment during abrasion. In contrast, U50F50, which incorporated a higher foil fraction, showed the lowest weight loss (14.02%), indicating improved abrasion resistance due to the rigidity of the metallic foil and reflective characteristics that enhance the structural stability of the composite.

The abrasion performance observed in the MSW-derived composites provides critical insight into their suitability for outdoor paving slab applications. The relatively low weight loss (<20%) after 60 s of accelerated abrasion indicates that these composites possess sufficient surface durability for low to medium mechanical stress environments. Compositions with higher foil bag ratios, ranging from 30 to 50 wt%, demonstrated the most stable performance, suggesting that U50F50 is the optimal formulation for outdoor applications where prolonged UV exposure and mechanical abrasion are prevalent.

### 3.8. Effects of Mixing Ratios and Weathering on Thermal Conductivity of the Samples

The thermal performance of the plastic-based composite panels manufactured from municipal solid waste (MSW) was analyzed to evaluate their potential for thermal insulation and heat transfer resistance for paving slab applications. Table 9 presents the mean values and standard deviations of thermal conductivity (λ) and thermal resistance (R) for all composite samples. The data indicate that both parameters vary significantly (*p* < 0.001) depending on the type and proportion of waste components incorporated into the structure of the composite. It was found that the measured thermal conductivity values ranged from 0.136 to 0.189 W/m·K, indicating that all of the composite samples exhibited relatively low heat transfer capability compared to that of typical conventional cementitious paving materials. This suggests that MSW-derived composites possess favorable thermal insulation characteristics, which could contribute to improved surface comfort and reduced heat retention in outdoor environments [8,21]. It can be seen from the U80P15A5 that displayed the lowest thermal conductivity (0.136 W/m·K), while U50F50 exhibited the highest value (0.189 W/m·K). The general trend indicates an increase in thermal conductivity with increasing foil content, which can be attributed to the intrinsic thermal properties of metallic aluminum. At lower foil ratios (U80P15A5), the composite structure is dominated by polymeric and paper-based constituents, both of which are poor conductors of heat. The trapped air within the fibrous paper layers and polymeric matrix increases interfacial phonon scattering and reduces heat transfer efficiency. Different ratios and layers of UHT carton, plastic bags, and aluminum foil are arranged as shown in Table 4. Conversely, increasing the foil bags proportion (U70F30 to U50F50) enhances the composite’s effective heat conduction pathway due to the high thermal conductivity of metallic particles embedded within the plastic matrix. This behavior confirms the dual role of aluminum foil as both a structural reinforcement and a thermal conductor, influencing the balance between mechanical durability and thermal insulation [14,51].

As expected, thermal resistance showed an inverse relationship to thermal conductivity. The thermal resistance values ranged from 0.142 to 0.213 m^2^·K/W, with U50F50 showing the highest R value (0.213 m^2^·K/W) despite its relatively high conductivity, due to its increased sample thickness and internal composite structure that contribute to additional resistance to heat flow. The composite samples with lower foil content (U80P15A5), including the control sample (U100), exhibited lower thermal resistance, primarily due to the higher porosity and weaker interfacial bonding caused by plastic degradation [6,52]. The higher void fraction and disrupted interfaces facilitate localized heat transfer, thus slightly decreasing R. For U70F30, U60F40, and U50F50 compositions, the progressive increase in foil layers reduced the total number of thermal interfaces and enhanced the continuity of heat conduction pathways through the matrix. A foil bag’s high intrinsic conductivity (approximately 205 W/m·K) significantly contributes to composite λ as its fraction increases. However, this enhancement is partially offset by the insulating plastic matrix and the non-uniform dispersion of foil fragments, which introduce micro-gaps that scatter phonons.

It is recognized that thermal behavior plays a vital role in determining surface comfort and long-term durability under solar exposure of slab products. Composite samples with lower thermal conductivity (U80P15A5 and U100) are advantageous for reducing heat accumulation, potentially mitigating urban heat island effects in outdoor installations. Conversely, composites with higher foil content, while slightly more conductive, offer enhanced thermal stability and structural integrity, making them suitable for load-bearing or high-durability applications [21,47,52].

## 4. Conclusions

This study comprehensively investigated the mechanical, physical, and thermal performance of polymer-based composites fabricated from MSW constituents, including UHT cartons, plastic bags, aluminum foil, and foil bags under both unweathered and accelerated weathering conditions. The experimental results and statistical analyses provide the following key conclusions:

The mixing ratios exerted a significant influence on the mechanical properties of the composites. Incorporation of foil bags at 30 to 50 wt% (U50F50) achieved the highest mechanical performance with MOR, MOE, CS, CM, and HN values of 121.20 MPa, 2.98 GPa, 28.56 MPa, 2.12 GPa, and 66.25, respectively. These findings demonstrate the reinforcing efficiency of foil bags and their ability to enhance composite stiffness and toughness. Accelerated weathering resulted in moderate reductions in all mechanical properties, primarily due to plastic chain scission, leaching of soluble components, and surface cracking. Nonetheless, the composites containing higher foil bag ratios exhibited superior resistance to environmental degradation, maintaining up to 90% of their initial strength after exposure to weathering. This suggests that metallic foil layers serve as UV-reflective and moisture-barrier phases that mitigate photooxidative and hydrolytic deterioration.

Increasing the foil bag content from 30 to 50 wt% effectively reduced both WA and TS values by up to 40%, attributable to the reduced number of free hydroxyl groups and the formation of compact, less permeable interfaces. The density of the composites increased with rising foil content, reaching a maximum of 1.05 g/cm^3^ for U50F50, primarily due to the higher intrinsic density of aluminum foil.

The abrasion tests confirmed that weight loss after 60 s ranged from 8.56% and 12.96% for unweathered samples and between 14.02% and 19.78% for accelerated weathering samples. Composites with higher foil content exhibited the lowest material loss, reflecting enhanced surface strength and cohesion. These results affirm the suitability of the U50F50 formulation for paving slabs exposed to repeated mechanical contact and environmental stress. Thermal analysis revealed that the thermal conductivity (λ) of the composites ranged from 0.136 to 0.189 W/m·K, while thermal resistance (R) ranged from 0.142 to 0.213 m^2^·K/W. Increasing foil fractions improved conductivity due to the metallic phase continuity but also enhanced structural stability. Thus, the composites can be tailored for specific applications where trade-offs between thermal insulation and mechanical durability are required.

**Figure 9:**
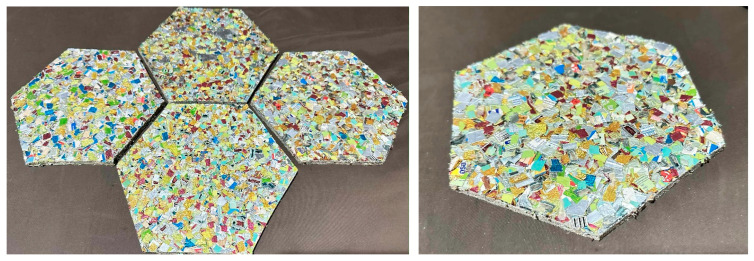
Paving slabs prototype based on optimal formulation (U50F50).

The experimental findings confirm that MSW-derived plastic composites, particularly those containing 50 wt% foil bags (U50F50), demonstrate a balanced combination of strength, durability, and thermal functionality. The integration of metallic and polymeric waste components not only enhances mechanical robustness and weathering resistance but also promotes circular material utilization by transforming mixed waste streams into value-added construction products. These properties collectively indicate strong potential for the development of sustainable paving slabs (Figure 9) with reduced environmental impact and extended service life.

## Figures and Tables

**Table 1:** Mixture ratios of plastic composites for this research.

Compositions	UHT Carton (wt%)	Plastic Bags (wt%)	Aluminum Foil (wt%)	Foil Bags (wt%)
U100	100	-	-	-
U80P15A5	80	15	5	-
U80P5A15	80	5	15	-
U70F30	70	-	-	30
U60F40	60	-	-	40
U50F50	50	-	-	50

**Table 2:** Mechanical properties of plastic composites with different municipal solid waste contents under accelerated weathering test.

Compositions (wt%)	Mechanical Properties											
	MOR	MOR (w)	MOE	MOE (w)	CS	CS (w)	CM	CM (w)	DSW	DSW (w)	HN	HN (w)
	(MPa)	(MPa)	(GPa)	(GPa)	(MPa)	(MPa)	(GPa)	(GPa)	(MPa)	(MPa)	(Shore D)	(Shore D)
U100	80.06	64.05	2.44	2.11	20.12	16.55	1.42	1.22	43.23	37.45	62.08	57.07
U80P15A5	64.96	52.22	2.32	1.99	19.69	14.87	1.02	0.89	40.12	35.12	60.07	55.90
U80P5A15	93.27	74.39	2.33	1.88	19.89	16.12	1.65	1.43	46.69	42.02	62.43	58.49
U70F30	109.5	88.23	2.52	2.19	22.45	19.02	1.89	1.66	48.03	44.65	63.56	60.33
U60F40	115.61	95.67	2.65	2.26	27.47	22.11	1.96	1.69	50.12	46.89	64.53	61.45
U50F50	121.20	100.44	2.98	2.54	28.56	24.58	2.12	1.84	54.25	49.04	66.25	63.17
*p*-value	0.000 *	0.000 *	0.001 *	0.001 *	0.000 *	0.000 *	0.001 *	0.001 *	0.000 *	0.000 *	0.000 *	0.000 *

Note: (w) samples under accelerated weathering test. * *p-value* less than 0.05 is considered significance.

**Table 3:** Physical properties of plastic composites with different municipal solid waste contents under accelerated weathering test.

Compositions (wt%)	Physical Properties					
	WA	WA (w)	TS	TS (w)	DS	DS (w)
	(%)	(%)	(%)	(%)	(g/cm^3^)	(g/cm^3^)
U100	2.74	4.95	2.90	5.60	0.94	0.85
U80P15A5	3.55	6.70	3.04	5.86	0.82	0.76
U80P5A15	3.01	5.43	2.95	5.66	0.96	0.88
U70F30	2.89	5.02	2.76	5.31	1.02	0.95
U60F40	2.56	4.56	2.06	3.97	1.03	0.97
U50F50	2.14	3.81	1.89	3.21	1.05	0.99
*p*-value	0.002 *	0.002 *	0.001 *	0.001 *	0.002 *	0.002 *

Note: (w) samples under accelerated weathering test. * *p-value* less than 0.05 is considered significance.

**Table 4:** Cross-section images of the composite surfaces with different UHT carton, plastic bags, aluminum foil, and foil bags contents.

**Components**	**U100 (100:0:0:0 wt%)**	**U80P15A5 (80:15:5:0 wt%)**	**U80P5A15 (80:5:15:0 wt%)**
UHT carton: Plastic bags: Aluminum foil: Foil bags:	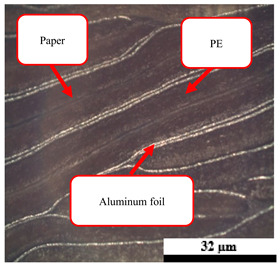	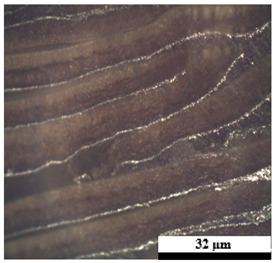	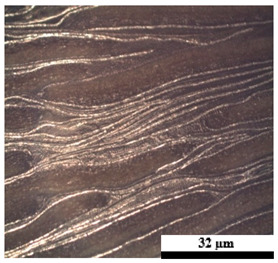
**Components**	**U70F30 (70:0:0:30 wt%)**	**U60F40 (60:0:0:40 wt%)**	**U50F50 (50:0:0:50 wt%)**
UHT carton: Plastic bags: Aluminum foil: Foil bags:	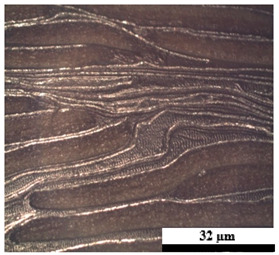	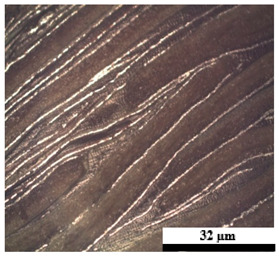	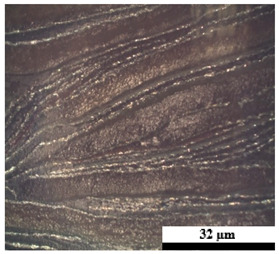

**Table 5:** Surface images of plastic composites with different UHT carton, plastic bags, aluminum foil, and foil bags contents.

**Components**	**U100 (100:0:0:0 wt%)**	**U80P15A5 (80:15:5:0 wt%)**	**U80P5A15 (80:5:15:0 wt%)**
UHT carton: Plastic bags: Aluminum foil: Foil bags:	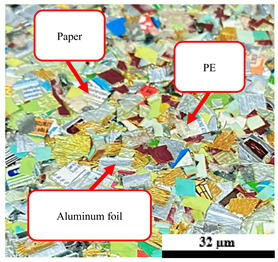	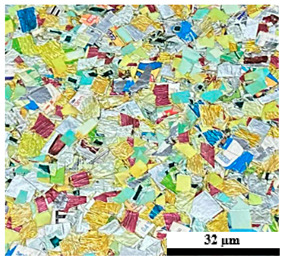	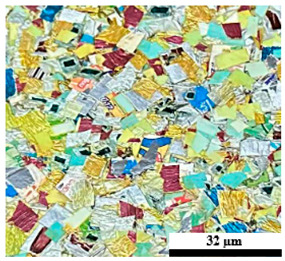
**Components**	**U70F30 (70:0:0:30 wt%)**	**U60F40 (60:0:0:40 wt%)**	**U50F50 (50:0:0:50 wt%)**
UHT carton: Plastic bags: Aluminum foil: Foil bags:	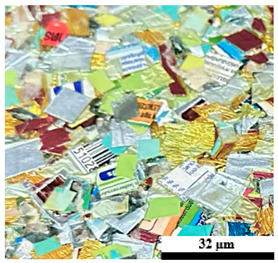	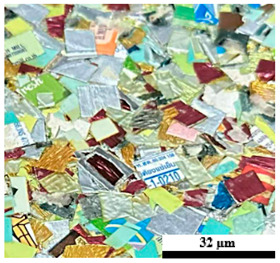	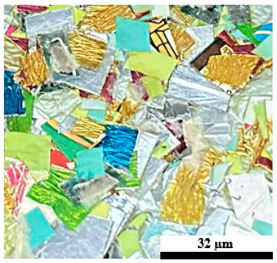

**Table 6:** Surface images of plastic composites with different UHT carton, plastic bags, aluminum foil, and foil bags contents under accelerated weathering test.

**Components**	**U100 (100:0:0:0 wt%)**	**U80P15A5 (80:15:5:0 wt%)**	**U80P5A15 (80:5:15:0 wt%)**
UHT carton: Plastic bags: Aluminum foil: Foil bags:	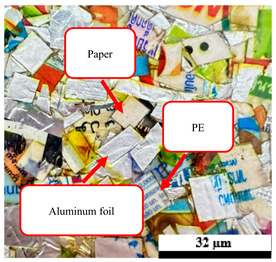	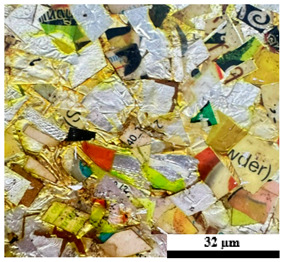	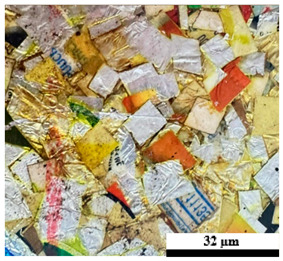
**Components**	**U70F30 (70:0:0:30 wt%)**	**U60F40 (60:0:0:40 wt%)**	**U50F50 (50:0:0:50 wt%)**
UHT carton: Plastic bags: Aluminum foil: Foil bags:	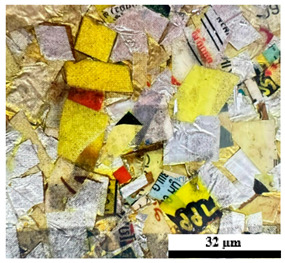	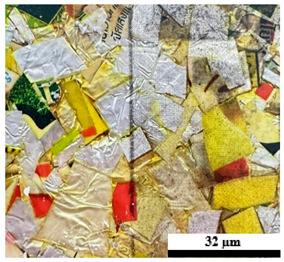	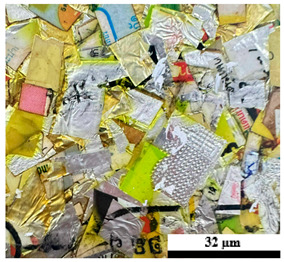

**Table 7:** Abrasion properties of plastic composites with different municipal solid waste content.

Code	Sample Weight (g)				Weight Loss
	Initial Weight	20 s	40 s	60 s	(%)
U100	22.657	22.567	21.345	20.356	10.156
U80P15A5	21.897	21.790	20.786	19.059	12.961
U80P5A15	21.809	21.715	20.369	19.457	10.785
U70F30	22.456	22.346	21.357	20.077	10.594
U60F40	22.344	22.266	21.055	20.112	9.989
U50F50	22.487	22.366	21.856	20.562	8.561
*p*-value	0.001 *	0.000 *	0.000 *	0.000 *	0.000 *

Note: * *p-value* less than 0.05 is considered significance.

**Table 8:** Abrasion properties of plastic composites with different municipal solid waste content under accelerated weathering test.

Code	Sample Weight (g)				Weight Loss
	Initial Weight	20 s	40 s	60 s	(%)
U100	22.204	21.890	20.278	19.135	16.039
U80P15A5	21.459	21.136	19.747	17.915	19.782
U80P5A15	21.373	21.064	19.351	18.290	16.856
U70F30	22.007	21.676	20.289	18.872	16.612
U60F40	21.897	21.598	20.002	18.905	15.827
U50F50	22.037	21.695	20.763	19.328	14.016
*p*-value	0.000 *	0.000 *	0.000 *	0.001 *	0.000 *

Note: * *p-value* less than 0.05 is considered significance.

**Table 9:** Thermal conductivity and thermal resistance of plastic composites with different municipal solid waste contents.

Code	Thermal Conductivity (λ) (W/m·K)		Thermal Resistance (R) (m^2^·K/W)	
	$\overset{-}{X}$	S.D.	$\overset{-}{X}$	S.D.
U100	0.148	(±0.003)	0.151	(±0.002)
U80P15A5	0.136	(±0.002)	0.142	(±0.002)
U80P5A15	0.149	(±0.001)	0.156	(±0.001)
U70F30	0.165	(±0.002)	0.187	(±0.003)
U60F40	0.175	(±0.001)	0.205	(±0.001)
U50F50	0.189	(±0.002)	0.213	(±0.002)
*p*-value	0.000 *		0.000 *	

Note: * *p-value* less than 0.05 is considered significance.

## Data Availability

The data presented in this study are available on request from the corresponding author due to this research is in the process of developing mixing ratios and product prototypes.
